# Hospital-acquired fever in oriental medical hospitals

**DOI:** 10.1186/s12913-018-2896-1

**Published:** 2018-02-07

**Authors:** Soo-youn Moon, Ki-Ho Park, Mi Suk Lee, Jun Seong Son

**Affiliations:** 1 Department of Internal Medicine, Kyung Hee University School of Medicine, Seoul, South Korea; 2 Division of Infectious Diseases, Department of Internal Medicine, Kyung Hee University Hospital at Gangdong, 892, Dongnam-ro, Gangdong-gu, Seoul, South Korea

**Keywords:** Fever, Hospital-acquired, Traditional oriental medicine, Etiology

## Abstract

**Background:**

Traditional Oriental medicine is used in many Asian countries and involves herbal medicines, acupuncture, moxibustion, and cupping. We investigated the incidence and causes of hospital-acquired fever (HAF) and the characteristics of febrile inpatients in Oriental medical hospitals (OMHs).

**Methods:**

Patients hospitalized in two OMHs of a university medical institute in Seoul, Korea, were retrospectively reviewed from 2006 to 2013. Adult patients with HAF were enrolled.

**Results:**

There were 560 cases of HAF (5.0%). Infection, non-infection, and unknown cause were noted in 331 cases (59.1%), 109 cases (19.5%), and 120 cases (21.4%) of HAF, respectively. Respiratory tract infection was the most common cause (51.2%) of infectious fever, followed by urinary tract infection. Drug fever due to herbal medicine was the most common cause of non-infectious fever (53.1%), followed by procedure-related fever caused by oriental medical procedures. The infection group had higher white blood cell count (WBC) (10,400/mm^3^ vs. 7000/mm^3^, *p* < 0.001) and more frequent history of antibiotic therapy (29.6% vs. 15.1%, *p* < 0.001). Multivariate analysis showed that older age (odds ratio (OR) 1.67, 95% confidence interval (C.I.) 1.08–2.56, *p* = 0.020), history of antibiotic therapy (OR 3.17, C.I. 1.85–5.41, *p* < 0.001), and WBC > 10,000/mm^3^ (OR 2.22, C.I. 1.85–3.32, *p* < 0.001) were associated with infection.

**Conclusions:**

Compared to previous studies on HAF in Western medicine, the incidence of HAF in OMHs was not high. However, Oriental medical treatment does play some role in HAF. Fever in patients with history of antibiotic therapy, or high WBC was more likely of infectious origin.

## Background

Fever is a common clinical event in hospitalized patients. Although fever is frequently suspected and proven to be related to infections, diverse etiologies may account for fever in hospitalized patients.

Hospital-acquired febrile illness is defined as a fever occurring at least 48 h after hospital admission [1]. The prevalence of hospital-acquired febrile illness has been estimated at 2% to 31% for medical inpatients [2]. There have been studies on the etiologies of fever in elderly patients, solid organ transplant recipients, cancer patients, and neutropenic hosts [3–7]. Fever was attributed to infection in 37% to 74% of patients, whereas a non-infectious etiology was identified in 3% to 52% of patients [1]. The most common infectious causes included urinary tract infection, pneumonia, sinusitis, and bloodstream infection [1]. The most common non-infectious causes were procedure-related, malignancies, and ischemic conditions [8].

Oriental traditional medicine has been practiced for a very long time in Korea, China, Japan and throughout Southeast Asia, including Vietnam, Thailand, and Tibet. It is also currently practiced as a part of alternative medicine in Western countries. Traditional Oriental medicine is part of medical practice in Korea, and there are many hospitals in which traditional Oriental medical doctors practice traditional medicine for hospitalized patients. In Korea, patients with cerebrovascular accident and elderly patients tend to seek oriental medical care more often than other patients [9].

Our medical centers are composed of a medical hospital, oriental medical hospital (OMH), and dental hospital. Many patients are hospitalized for traditional medical treatment, and some patients develop fever. When patients in the OMH develop fever, many are referred to the medical hospital for evaluation and treatment of fever. There has been no data on the incidence or etiology of fever in these patients. As far as we know, this is the first study on hospital-acquired febrile illness in OMHs.

This study was designed to identify the characteristics of febrile inpatients, causes of fever, and clinical outcomes of fever in OMHs and to identify the risk factors of febrile illness of infectious cause in these patients.

## Methods

This retrospective study was performed at two OMHs of a university medical institute in Seoul, Korea. The medical institute consists of two medical hospitals, two OMHs, and two dental hospitals. The study protocol was approved by institutional review board (IRB) of Kyung Hee University Hospital at Gangdong (IRB No. 2016–03-008). Informed consent from the patients was waived by IRB.

Patients hospitalized in the OMHs from June 2006 to June 2013 were retrospectively reviewed via electronic medical records by two infectious diseases specialists. Patients age 18 years or older were screened. Adult patients with axillary body temperature ≥ 37.8 °C after 48 h of hospitalization were enrolled. If a patient was transferred from another OMH where he or she was admitted for more than 48 h and where fever started within 48 h of hospitalization, these fevers were also considered hospital-acquired. If a patient was transferred from a medical hospital or long-term care facility and fever had started within 48 h of hospitalization to the OMH, he or she was excluded.

Demographic characteristics, clinical features, laboratory data, and treatment history were collected by review of medical records. Infection was defined using criteria proposed by the Centers for Disease Control and Prevention [10].

Fever was considered procedure-related if there was a transient temperature elevation during the 48 h period following an invasive procedure and no evidence of infection elsewhere [1]. Drug fever was defined by diagnostic criteria adapted from those described by Young et al. [11] Patients with fever accompanied by drug rash were included if the fever resolved after discontinuing the offending drug. If patients with acute intra-cranial hemorrhage (ICH) or acute cerebral infarction developed fever with no other certain fever focus, the fever was considered related to the ICH or cerebral infarction. If patients had massive gastro-intestinal bleeding and no other obvious fever focus, the fever was considered related to gastrointestinal bleeding. If a fever was observed after transfusion of blood products such as packed RBC or platelet concentrates and no other cause was found, the fever was considered related to the transfusion. If a fever was observed in a severely dehydrated patient and it disappeared after adequate hydration, the fever was considered to be caused by dehydration. When there was unexplained fever in advanced cancer patients or patients with hematologic malignancy, the fever was considered a cancer fever.

If the etiology was uncertain or data was insufficient to determine a cause, the fever was defined as unknown. Defervescence was defined as peak body temperature below 37.3 °C for more than 2 days.

McCabe classification was used to evaluate the severity of underlying illnesses [12].

SPSS for Windows version 11.0 (SPSS Inc., Chicago, IL, USA) was used for statistical analysis. Student’s t-test and Chi-square test were used for univariate analysis, and a logistic regression test was used for multivariate analysis. Factors with statistical significance in univariate analysis were chosen for multivariate analysis. *P* values < 0.05 were considered significant.

## Results

A total of 11,207 adult patients were hospitalized in the OMHs during the study period. Among those 11,207 patients, 560 cases with hospital-acquired febrile events were identified (5.0%).

Infection was identified as the cause of fever in 331 cases (59.1%). In 179 cases (32.0%), non-infectious causes of fever were identified (Table 1). There were 50 cases (8.9%) of fever with unknown etiology. Among patients with fever caused by infections, respiratory tract infection was the most common cause (166 cases, 51.2%), followed by urinary tract infection (99 cases, 30.6%) and intra-abdominal infection (39 cases, 12.1%) (Table 1). Among patients with respiratory infection, aspiration pneumonia was the most common cause. Among patients with non-infectious fever, drug fever was the most common cause (101 cases, 56.4%), followed by procedure-related fever (24 cases, 13.4%) and cancer fever (23 cases, 12.8%). Among 101 patients with drug fever, herbal medicine was the most common cause (95 cases, 94.1%), while antibiotics or bisphosphonate were the cause of drug fever in 6 patients.

**Table 1:** Etiology of Hospital-acquired Fever in OMHs

Category	No. (%)
Infection	331 (59.1)
Respiratory tract	166 (51.2)
Urinary tract	99 (30.6)
Intra-abdominal	39 (12.1)
Skin and soft tissue	18 (3.2)
Primary bacteremia/fungemia	10 (3.1)
Central nervous system	2 (0.6)
Bone & joint	2 (0.6)
Other	2 (0.6)
Non-infection	179 (32.0)
Drug fever	101 (56.4)
Post-procedure	24 (13.4)
Cancer fever	23 (12.8)
Transfusion related	7 (3.9)
Dehydration related	3 (1.7)
ICH related central fever^a^	2 (1.1)
Other	19 (10.6)
Unknown	50 (8.9)

^a^*ICH* intracranial hemorrhage

Among 11,207 adult patients hospitalized in OMHs, 10,880 were treated with herbal medicine, and 8125 underwent oriental procedures. There were 8040 patients who received acupuncture, 6585 patients who received moxibustion, and 4267 patients who received cupping. Ninety-five patients developed fever due to herbal medicine, 8 patients developed fever due to acupuncture, 7 developed fever patients due to moxibustion, and 1 patient developed fever due to cupping (Table 2). Overall incidence of procedure-related fever caused by invasive oriental procedures is 2.9% (16 episodes), while incidence of procedure-related fever caused by western medical procedures is 1.4% (8 episodes).

**Table 2:** Incidence of drug fever and procedure-related fever among patients receiving herbal medicine and/or oriental procedures among patients admitted to OMHs (*n* = 11,207)

Risk factor	No. of patients with risk factors	No. of patients with risk factors who developed fever	Percentage
Herbal medicine	10,880	95	0.87
Invasive oriental medical procedures	8125	16	0.20
Acupuncture	8040	8	0.10
Moxibustion	6585	7	0.11
Cupping	4267	1	0.02

Table 3 shows the patient characteristics of febrile patients in the OMHs. The mean age of these patients was 61.4 ± 15.2 years, and 49.6% of the patients were male. Patients with fever of infectious origin (infection group) were older than patients with fever of non-infectious origin (non-infection group) (63.7 ± 15.3 years vs. 58.0 ± 14.7 years, *p* < 0.001). More patients in the infection group had history of CVA (54.1% vs. 35.2%, *p* < 0.001). There were more patients with malignancies in the non-infection group (55.9% vs. 32.9%, *p* < 0.001). There was no statistical difference in the frequency of previous admission history between the two groups. Among 560 patients with fever, 506 (90.4%) were treated with herbal medicine. Invasive oriental procedures, such as acupuncture, moxibustion, and cupping, were performed in 532 patients (95.0%). Patients in the infection group had more frequent history of antibiotic treatment before fever onset (29.6% vs. 15.1%). There was no significant difference in peak body temperature or fever duration between the infection group and non-infection group. WBC count was higher in the infection group (median 10,400/mm3 vs. median 7000/mm3, *p* < 0.001); however, CRP level was not different between the two groups (Table 3).

**Table 3:** Characteristics of Patients with HAF in OMH by Etiology

	Total (*n* = 560)	Infection (*n* = 331)	Non-infection (*n* = 179)	*p*
Demographics				
Age (years)	61.4 ± 15.2	63.7 ± 15.3	58.0 ± 14.7	< 0.001
Male sex	278 (49.6)	169 (51.1)	81 (45.3)	0.228
Underlying diseases				
Diabets mellitus	141 (25.2)	90 (27.2)	40 (22.3)	0.243
Hypertension	234 (41.8)	161 (48.6)	60 (33.5)	0.001
Cerebrovascular accident	251 (44.8)	179 (54.1)	63 (35.2)	< 0.001
Solid cancer	246 (43.9)	109 (32.9)	100 (55.9)	< 0.001
Metastatic cancer	205 (36.6)	88 (26.6)	85 (47.5)	< 0.001
Healthcare- associatied conditions				
Receipt of anticancer chemotherapy	113 (20.2)	40 (12.1)	56 (31.3)	< 0.001
Central venous catheter	68 (12.1)	43 (13.0)	21 (11.7)	0.682
Urinary catheter	111 (19.8)	79 (23.9)	27 (15.1)	0.022
Percutaneous drainage	68 (12.1)	47 (14.2)	20 (11.2)	0.410
Previous operation	76 (13.6)	47 (14.2)	21 (11.7)	0.434
Previous intervention	24 (4.3)	14 (4.2)	9 (5.0)	0.678
Previous admission	414 (74.0)	250 (75.5)	134 (74.9)	0.867
Previous antibiotics	128 (22.9)	98 (29.6)	27 (15.1)	< 0.001
Concurrent treatment				
Herbal medicine	506 (90.4)	310 (93.7)	161 (89.9)	0.132
Invasive Oriental medical procedure	532 (95.0)	314 (94.9)	171 (95.5)	0.739
Acupuncture	502 (89.6)	302 (91.2)	161 (89.9)	0.630
Cupping	97 (17.3)	56 (17.0)	35 (19.6)	0.468
Moxibustion	417 (74.5)	242 (73.3)	138 (77.1)	0.352
SIRS^a^ (severe or shock)	47 (8.4)	28 (8.5)	14 (7.8)	0.788
Laboratory findings at the onset of fever				
White Blood Cell(/mm^3^)	9550 (100–32,500)	10,400 (800–32,500)	7000 (100–24,000)	< 0.001
Neutropenia (WBC < 1000/mm^3^)	5 (0.9)	3 (1.0)	2 (1.3)	0.662
C-reactive protein	5.3 (0.0–39.7)	5.4 (0.0–39.7)	4.2 (0.0–22.5)	0.767
Fever pattern				
Peak body temperature (°C)	38.5 ± 0.6	38.6 ± 0.6	38.4 ± 0.6	0.151
Duration of fever	1.0 (0.0–18.0)	1.0 (0.0–18.0)	1.0 (0.0–17.0)	0.263
No defervescence	43 (7.7)	23 (6.9)	12 (6.7)	0.917

^a^*SIRS* systemic inflammatory response syndrome

More patients with infectious fever received consultations for evaluation and management of fever (72.8% vs. 26.3%, *p* < 0.001). Antibiotics were prescribed in 302 cases (53.9%). More patients in the infection group were treated with antibiotics (73.7% vs. 25.1%, *p* < 0.001), and the duration of antibiotic therapy was also longer in the infection group (median 9 days vs. median 5.5 days, *p* = 0.002). Overall 30-day mortality rate was 9.6% (Table 4).

**Table 4:** Management and clinical outcome of febrile patients in OMH

	Total (n = 560)	Infection (n = 331)	Non-infection (n = 179)	*p*
Medical consult	297 (53.0)	241 (72.8)	47 (26.3)	< 0.001
Antibiotics	302 (53.9)	244 (73.7)	45 (25.1)	< 0.001
Duration of antibiotics	8.0 (0.0–40.0)	9 (1–40)	5.5 (1–21)	0.002
Antipyretics	112 (20.0)	72 (21.8)	31 (17.3)	0.339
Surgical treatment	7 (1.3)	3 (0.9)	4 (2.2)	0.248
30-day mortality	46/477 (9.6)	21 (7.6)	14 (8.9)	0.616

Table 5 shows multivariate analysis for risk factors associated with infectious origin in febrile illnesses. Patients age over 65 years developed fever of infectious origin 1.666 times more often (95% confidence interval (C.I.); 1.082–2.564, *p* = 0.020) than younger patients. When patients had a history of antibiotic use, fever of infectious origin was 3.166 times more likely (95% C.I.; 1.852–5.413, *p* < 0.001). Fever in the patients with WBC higher than 10,000/mm^3^ were 2.223 times more likely to be caused by infection (95% C.I.; 1.486–3.324, *p* < 0.001). In patients with cancer or history of chemotherapy, the chance of infectious fever decreased 0.263 times (95% C.I.; 0.116–0.594, *p* = 0.001) and 0.507 times (95% C.I.; 0.289–0.0887, *p* = 0.017), respectively.

**Table 5:** Factors associated with infectious origin in febrile illness in OMHs

	Univariate analysis	*p*	Multivariate analysis	*p*
	OR (95% C.I.)		OR (95% C.I.)	
Age > 65	2.250 (1.544–3.277)	< 0.001	1.666 (1.082–2.564)	0.020
Hypertension	1.878 (1.287–2.741)	0.001		
Cerebrovascular accident	2.168 (1.490–3.156)	< 0.001		
Urinary catheter	1.765 (1.091–2.855)	0.021		
Previous antibiotic	2.368 (1.475–3.798)	< 0.001	3.166 (1.852–5.413)	< 0.001
WBC > 10,000	3.730 (2.415–5.760)	< 0.001	2.223 (1.486–3.324)	< 0.001
Solid cancer	0.330 (0.232–0.468)	< 0.001	0.263 (0.116–0.594)	0.001
Metastatic cancer	0.347 (0.243–0.495)	< 0.001		
Recipient of anticancer chemotherapy	0.294 (0.191–0.452)	< 0.001	0.507 (0.289–0.0887)	0.017

## Discussion

Oriental traditional medicine in East Asia, including Korea, China, and Japan, uses acupuncture, moxibustion, cupping, herbal medicines, and manual therapies [13]. In Korea, traditional Oriental medicine is called Hanbang and is an inseparable component of Korean culture and Korean medical services [9]. Traditional Oriental medicine is included in the national health care system in Korea. National policies have been developed based on its historical and cultural background in a way that differs from its use as a complementary and alternative medicine in Western society [14]. Korea has the highest percentage (15.3%) of traditional Oriental medical doctors in hospitals and clinics in East Asia, followed by mainland China (12.6%) and the Taiwan region (9.7%) [14] Korean patients with cerebrovascular accidents (ICH or cerebral infarction), advanced cancer, facial palsy, and old age tend to seek traditional Oriental medicine more often than patients with other acute illnesses [9]. Because traditional Oriental medicine uses different treatment modalities than Western medicine, the etiology of fever might be different. As far as we know, this is the first study on the incidence and etiologies of hospital-acquired fever in patients hospitalized at OMHs.

In this study, incidence of HAF was 5.2%, lower than that of other studies [2, 15]. Infection accounted for 59.1% of HAF among patients hospitalized in OMHs. In Arbo’s study of HAF, infection accounted for 56.0% of fever, which is similar to our results [1]. Chang’s study on liver transplant recipients showed a higher incidence of infection (78.0%) [3]. In cancer patients, infection, non-infectious causes, and fever of unknown origin represented 67.0%, 23.0%, and 10.0% of cases, respectively [7]. The respiratory tract was the most frequently involved site in cancer patients, [7] similar to our results.

In OMHs, more patients with solid cancer (55.9% vs. 32.9%, *p* < 0.001) and history of anti-cancer chemotherapy (31.3% vs. 12.1%, *p* < 0.001) have non-infectious fever. In these patients, moxibustion was more commonly used, and herbal medication was less commonly prescribed without statistical significance. Moxibustion was more commonly used in the non-infection group, while herbal medicine was more frequently prescribed to patients in the infection group. There were more patients with cancer in the non-infection group than in the infection group. Moxibustion was more frequently used in the non-infection group for pain relief in cancer patients. Some studies have shown that moxibustion is effective in reducing pain associated with osteoarthritis and herpes zoster [16]. Herbal medicine is a main treatment modality in traditional Oriental medicine and is prescribed to most patients. Unlike Western medicine, Oriental herbal medicine can only be administered by oral route. Some patients with advanced cancer were unable to orally take medication, so herbal medicine could not be prescribed.

Drug fever accounted for 56.4% of non-infectious fever in our study. In Cunha’s studies on fever in a neurosurgical intensive care unit and intensive care unit, drug fever occurred in approximately 10.0% of patients [17, 18]. In Toussaint’s study on fever in cancer patients, drug fever accounted for 18.0% of fever not attributed to infection (non-infectious fever and fever of unknown origin together), which is lower than our study result [7]. In Oisumi’s study on drug fever caused by antibiotics, drug fever was recognized in 13.1% of 390 patients receiving parenteral antibiotic therapy for pulmonary infections. Drugs have been estimated to cause 10.0–15.0% of adverse events and 3.0–5.0% of drug fever in hospitalized patients in the US [19].

Procedure-related fever in Western medicine was about 1.5% to 5.9%, [2, 7] while incidence of procedure-related fever in our OMH was 4.2%, which was not different from other studies. Among the procedure-related fever in our OMH, fever related to invasive oriental procedure was higher than that related to western medical procedures (2.9% vs. 1.4%).

Old age, history of antibiotic use, and high WBC were associated with infection. Peak body temperature was not associated with infection in this study. In Trivalle’s study on hospital-acquired febrile illness in the elderly, the number of invasive procedures preceding a febrile episode was a significant predictor of infection [2]. Other studies have shown that higher maximum temperature or higher peak WBC was associated with an infectious etiology for fever [8].

This study has some limitations. First, it was a retrospective study performed in OHMs. Culture and imaging studies are not performed in every patient with HAF. Therefore, some patients with infection may not have been identified and classified with unknown fever. Second, this study was performed at two OMHs of a university teaching hospital. In our hospitals, patients with advanced cancer or cerebrovascular attack are common. Patient characteristics and severity of underlying illness may be different from those of other OMHs.

## Conclusions

In this study, incidence of HAF was not higher in OMHs, [2, 15] and infection was the most common cause of HAF. Fever in patients with history of antibiotic treatment and high was more likely of infectious origin.

Herbal medicine and invasive oriental medical procedures do play some role in HAF as herbal medicine was the most common cause of drug fever and invasive oriental medical procedures caused procedure related fever more frequently than Western medical procedures in OMHs.

To identify the etiology of HAF in OMHs and the risk factors of fever of infectious origin, further multicenter study is suggested.

## Abbreviations

C.I.: Confidence interval
CRP: C-reactive protein
HAF: Hospital-acquired fever
ICH: Intra-cranial hemorrhage
IRB: Institutional review board
OMH: Oriental medical hospital
OR: Odds ratio
RBC: Red blood cell
WBC: White blood cell count
